# Real Time Multiplicative Memory Amplification Mediated by Whole-Cell Scaling of Synaptic Response in Key Neurons

**DOI:** 10.1371/journal.pcbi.1005306

**Published:** 2017-01-19

**Authors:** Iris Reuveni, Sourav Ghosh, Edi Barkai

**Affiliations:** Sagol Department of Neurobiology, Faculty of Natural Sciences, University of Haifa, Haifa, Israel; Research Center Jülich, GERMANY

## Abstract

Intense spiking response of a memory-pattern is believed to play a crucial role both in normal learning and pathology, where it can create biased behavior. We recently proposed a novel model for memory amplification where the simultaneous two-fold increase of all excitatory (AMPAR-mediated) and inhibitory (GABA_A_R-mediated) synapses in a sub-group of cells that constitutes a memory-pattern selectively amplifies this memory. Here we confirm the cellular basis of this model by validating its major predictions in four sets of experiments, and demonstrate its induction via a whole-cell transduction mechanism. Subsequently, using theory and simulations, we show that this whole-cell two-fold increase of all inhibitory and excitatory synapses functions as an instantaneous and multiplicative amplifier of the neurons’ spiking. The amplification mechanism acts through multiplication of the net synaptic current, where it scales both the average and the standard deviation of the current. In the excitation-inhibition balance regime, this scaling creates a linear multiplicative amplifier of the cell’s spiking response. Moreover, the direct scaling of the synaptic input enables the amplification of the spiking response to be synchronized with rapid changes in synaptic input, and to be independent of previous spiking activity. These traits enable instantaneous real-time amplification during brief elevations of excitatory synaptic input. Furthermore, the multiplicative nature of the amplifier ensures that the net effect of the amplification is large mainly when the synaptic input is mostly excitatory. When induced on all cells that comprise a memory-pattern, these whole-cell modifications enable a substantial instantaneous amplification of the memory-pattern when the memory is activated. The amplification mechanism is induced by CaMKII dependent phosphorylation that doubles the conductance of all GABA_A_ and AMPA receptors in a subset of neurons. This whole-cell transduction mechanism enables both long-term induction of memory amplification when necessary and extinction when not further required.

## Author Summary

Amplifying the strength of a neuronal assembly that underlies a behavioral choice can lead to a particularly long lasting dominant memory. We report experimental and theoretical evidence for a long-term mechanism that amplifies the response of a neuronal assembly which we termed “memory amplification mechanism”. The amplification mechanism is mediated by doubling the strength of all inhibitory and all excitatory synapses in the cell and is induced by whole-cell phosphorylation of all inhibitory and excitatory synaptic receptors in a subset of cells, via a process that is distinct from memory formation. Computationally, the inherent scaling of both excitation and inhibition yields a robust and stable amplifier of the neuron’s response. When such an amplifier is induced in a set of cells that compose a memory-pattern, it can selectively amplify the response of this memory. The memory amplification mechanism is independent from associative learning. Thus, while associative learning forms a memory that encodes new associations, the amplification mechanism can promote an already formed memory to a dominant memory.

## Introduction

Traditionally, increased synaptic response is believed to reflect activity dependent synaptic plasticity, in which the association between different inputs is strengthened through synaptic-specific potentiation. We have previously shown that acquiring the skill to perform in a particularly difficult olfactory-discrimination task [1–4] results in a robust enhancement of excitatory and inhibitory synaptic connectivity to and within the piriform cortex that lasts for days after training [5–8]. The synaptic enhancement was observed few days after the rats were last trained and thus indicates long term induced synaptic modifications.

In particular, we suggested that this increase in synaptic strength results from a process where in a subset of cells all AMPA and GABA_A_ receptors double their strength through a whole-cell two-fold increase of their single channel conductance. This process results in a two-fold increase in the strength of all excitatory and inhibitory synapses in the cell [9], and thus can support the previously observed increase in synaptic strength [5–8]. Using a preliminary study of a computational network model [9] we suggested that if this mechanism is induced in a group of cells that compose a memory-pattern, it can substrate as a memory specific amplification mechanism [9]. A memory amplification mechanism should largely increase the spiking response of the cells that compose the memory pattern when the memory pattern is active, and have a minor effect when not active. In this work we aim to computationally establish the role of this mechanism in memory amplification by studying its effect on the net synaptic current and on the spiking response of a single cell. Moreover, through characterization of the amplification properties such as speed and stimulus dependencies we aim to obtain a further characterization of the functional effect of this mechanism.

The memory amplification model extends beyond the well-studied activity-dependent synaptic plasticity that was suggested to underlie memory formation in several key ways: (1) It amplifies the response of an already formed memory-pattern rather than forming a new association between the different inputs. (2) The increased synaptic excitation that underlies the memory amplification is not synapse specific and is mediated by the increased strength of virtually all the excitatory synapses in the cell. (3) The increase in synaptic strength is not mediated by an increased number of AMPA receptors, as was shown for LTP [10–12], but rather is mediated by increased AMPA receptor channel conductance. (4) Importantly, unlike activity-dependent synaptic plasticity, memory amplification requires a parallel and similar increase in the strength of synaptic inhibition. These characteristics enable this mechanism to be independent of memory formation and thus enable the promotion of an already learned memory into a dominant memory.

Several studies have shown that CaMKII-mediated phosphorylation of the AMPA channel in ser831 causes a two-fold increase in AMPA channel conductance [13–15], which is similar to the increase found in our setting. A co-increase in excitation and inhibition is vital for whole-cell balanced amplification. This suggests that both increased excitation and increased inhibition are induced by a shared process for which CaMKII is a potential candidate that doubles the conductance of all AMPA and GABA_A_ receptors in the cell. Indeed we recently showed that the increase in inhibitory and excitatory synaptic transmission that was induced by learning the task is CaMKII dependent and that this increase is the result of increased conductance of the GABA_A_ and AMPA channels [7, 8]. In these studies the blocker effect on each cell was measured by comparing the averaged synaptic event amplitude before and after the CaMKII blocker was applied. In the current study we further examined whether the task learning induced reduction in averaged synaptic event amplitude is mediated through the effect on memory amplification mechanism, namely through reversal of the two-fold multiplication of the strength of all GABA_A_ and AMPA mediated events in a subset of cells.

In these studies, miniature inhibitory and excitatory post-synaptic currents were recorded in trained and control animals before and after application of CaMKII blockers [7, 8]. The generation of a miniature synaptic event is triggered by a spontaneous release of one vesicle in the presynaptic site which activates synaptic receptors that are densely packed in a nanodomain cluster opposite to the release site [16]. Each nanodomain cluster is 70 nm in length, contains 20 synaptic receptors and has an average distance of 250 nm from other nanodomain clusters in the same PSD [16]. The binding of the CaMKII molecule to the PSD in the cytoplasmatic site [17, 18] enable it to phosphorylate the synaptic receptors. The nanoscale size of the cluster and the auto-phosphorylation characteristics of CaMKII can enable a single CaMKII molecule located in close proximity to phosphorylate all synaptic receptors in this cluster [19], and thus to affect all synaptic receptors that mediate a single miniature synaptic event.

The miniature synaptic events are spontaneously evoked and therefore do not reflect a pathway-dependent activity. This enables us to use these recordings to analyze the whole-cell effect of CaMKII and to study if this enhancement can be explained within the scope of the memory amplification mechanism. Moreover, while in the previous study [9] we indirectly inferred the multiplication mechanism based on between-groups comparison, in the current study, the learning specific effect of CaMKII blocker allows us to directly establish the induction of the memory amplification mechanism based on within-cell comparison. This enables us to further examine the segregated effect of learning and the induction of a cell-based two-fold multiplication through a whole-cell transduction mechanism.

If CaMKII phosphorylation is indeed the molecular mechanism that underlies memory amplification, it is expected that in the selected subset of cells CaMKII blocker will induce a two-fold reduction in all AMPA and GABA_A_ mediated synaptic responses, through a two-fold decrease in the conductance of all AMPA and GABA_A_ receptors. Thus the memory amplification model has four testable predictions for both GABA_A_ and AMPA mediated miniature synaptic events: (a) the effect of the CaMKII blocker will be prominent in a sub-group of cells. (b) In this subset of cells the effect will be mediated by a decrease in the single channel conductance. (c) The effect of the CaMKII blocker should correspond to a reduction of each event amplitude by a factor of two, and (d) the sub-group of cells that was affected by CaMKII blockers should be the sub-group of cells that was affected by task learning.

In this work we first analyze the experimental data in depth, to test the predictions of the model. Then, using theory and single-cell biophysical simulations, we show how such a whole-cell increase in excitatory and inhibitory synaptic inputs can function as an instantaneous and linear amplifier of the cell spiking response. These findings establish the basis for the memory amplification mechanism; namely, applying a cell specific linear amplifier on a subset of cells that constitutes a memory-pattern can lead to amplification of this memory when activated.

## Results

### Confirming the model predictions

#### Summary of the experimental procedure

The analysis was performed on data taken from sets of experiments that were published earlier [7, 8]. Here we present a short summary of these experiments, where a more detailed description can be found in the Material and Methods section or published before [7,8].

Water deprived rats were trained in an odor discrimination maze that consisted of 4 arms, where at each trial one randomly chosen arm contained water at its far end. Rats in the trained group had to choose the water containing arm based on a positive odor cue which signified the presence of water in the containing arm and negative odor cue which signified no water in the containing arm. The task was difficult to learn, as it took the rats 7–8 days to reach the learning criterion of the first odor pair (negative and positive odors). The training was terminated once the rats demonstrated enhanced learning capabilities using a different odor pair, which in average lasted 1–2 days only. Rats designated to the pseudo-trained group were rewarded with water when choosing any odor in a random manner and their exposure time to the odor maze was artificially matched with the rats of the trained group. Rats in the naive group were left in their home-cages and were water deprived only. 4–5 days after training termination, in which rats stayed in their home cage, whole-cell recordings were made from identified pyramidal cells in slices taken from the piriform cortex. In these recordings spontaneously evoked miniature synaptic events could be identified. Each cell exhibited only excitatory miniature postsynaptic synaptic currents (mEPSCs) or inhibitory synaptic currents (mIPSCs), due to a different pharmacology, holding potential and pipette solution (Materials and Methods).

#### CaMKII induces a whole-cell two fold increase of the GABA_A_ single channel current in a subset of cells from trained rats

We first tested the four predictions of the memory amplification model by analyzing the spontaneous miniature inhibitory synaptic events before and after the application of two different CaMKII blockers (KN93 and tatCN21) [7, 8].

**Fig 1:**
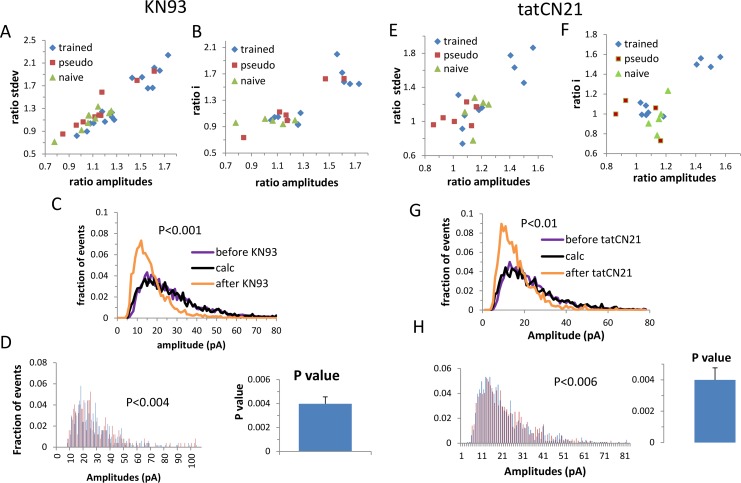
CaMKII blockers (KN93 left; tatCN21 right) act through a whole-cell, two-fold division of the GABA_A_ single channel conductance in a subset of cells from trained rats. A, E: Depicted for each cell is the effect of CaMKII blocker on the average and standard deviation of the event amplitudes. The effect of the CaMKII blocker clearly exhibits two clusters, where one cluster exhibits a large blocker effect on the average and standard deviation of the event amplitude (KN93: trained, n = 16; pseudo, n = 8; naïve, n = 10. tatCN21: trained, n = 10; pseudo, n = 7; naive, n = 5) B, F: Similarly, the effect of CaMKII blocker on the average event amplitude and single channel current exhibits two clusters (KN93: trained, n = 12; pseudo, n = 6; naive, n = 5. tatCN21: trained, n = 10; pseudo, n = 4; naïve, n = 5). C, G: An amplitude distribution curve averaged for all cells in the affected cluster (all cells in the blocker affected cluster for which the single channel current could be calculated; KN93: n = 5; tatCN21: n = 4) is shown for the event amplitudes recorded before (purple) and after (orange) CaMKII blocker. The blocker effect was computationally reversed (black curve) by applying the reverse calculation on the data recorded after applying the CaMKII blocker: The fraction of events that were affected by the drug was calculated (Materials and Methods). This fraction of events was randomly selected from the events recorded after the blocker was applied and the amplitude of these events was multiplied by a factor of two. A new amplitude distribution curve was calculated from the whole set of data, using both the multiplied and the non-multiplied events, exhibiting a fully computationally reversed CaMKII blocker effect. The P values were calculated using Cramer Von Mises two-sample test. D, H: the blocker effect could be reversed in each cell separately. Left: an example of one cell where the histogram of events amplitudes before CaMKII blocker (blue) matched the histogram of the calculated events amplitude after the blocker effect was computationally reversed (red), right the P value was calculated for each cell and the average of these P values was calculated from all cells in the highly affected cluster.

We started by testing the first prediction, namely that the effect of the CaMKII blocker on the inhibitory synaptic transmission would be prominent in a subset of cells. The effect of CaMKII blocker was calculated as the average amplitude of all the events that were recorded before CaMKII blocker was applied divided by the averaged amplitude of all events recorded after the blocker was applied. Since a multiplicative process equally affects the average and the standard deviation, for each cell we also measured the effect on the standard deviation of events’ amplitudes in order to yield a robust measure of the blocker effect. Plotting the effect of CaMKII blockers on the average and the standard deviation for each cell (Fig 1A and 1E) yielded a clear clustered effect (mean silhouette values: KN93; 0.72; tatCN21: 0.7) where the affected cluster exhibited a large CaMKII blocker effect (KN93: 165±9%, tatCN21: 147±7%) and the non-affected cluster exhibited a small effect (KN93:110±10%, tatCN21: 107±7%). Importantly, the effect of the CaMKII blockers differed significantly between neurons from the control (naïve and pseudo trained) and trained rats. While only a small fraction of the cells from the two control groups was in the affected cluster (KN93: 10%, tat: 0%) both CaMKII blockers had a pronounced effect on a large proportion of neurons taken from trained rats; 41% from the kn93-treated trained cells and 30% from the tatCN21-treated trained cells were clearly distinct from the remainder of the trained group and the control groups, which did not differ from each other. This clustered effect of the CaMKII blockers does not reflect animal-dependent variability, since for almost every cell in the affected cluster there was another cell from the same animal that belonged to the less affected cluster (6 out of 7 for KN93, and 4 out of 4 for the tatCN21). This analysis confirms our first prediction in which learning the task exerts its main effect on a subset of cells. A complete reversal of the learning effect in the affected cells requires that CaMKII blocker will reduce the averaged event amplitude by a factor of two. However, since in order to be in the physiological range we used moderate concentrations of CaMKII blocker, we did not attain a full blocker effect, where KN93 had clearly a bigger effect than tatCN21 (P<0.05). The synaptic structure enables a single CaMKII molecule to phosphorylate all the synaptic receptors that mediates a single miniature event (see introduction), therefore blocking the locally bound CaMKII will effectively reduce the amplitude of the miniature synaptic event by a factor of two. Thus due to the partial blocker effect a fraction of the events will reduce their amplitude by a factor of two, while the remainder of the events will not be affected. Calculating the fraction of events that were affected by the blocker (see Materials and Methods) in the affected cells yielded that 65% of the events were affected by KN93 and 47% of the events were affected by tatCN21.

To examine the second prediction we tested if CaMKII blockers also exhibited a clustered effect on the single channel conductance. We also tested if the reduction in the single channel current both overlapped with and was similar to the reduction in the average amplitude. We found that the effect of both CaMKII blockers (KN93 and tatCN21) on the single channel conductance was not homogenous over all cells in the trained group (mean silhouette values: KN93 0.8; tatCN21: 0.73), where some cells exhibited the same effect as the control groups while others exhibited a major effect (Fig 1B and 1F). Cells that exhibited a large decrease in the average amplitude exhibited a large decrease in the GABA_A_ single channel current (KN93: 167±15%, tatCN21: 152±4%) which was similar to the effect on the average event amplitude. These results led us to conclude that the effect of the CaMKII blocker on the average event amplitude was mediated by its effect on single channel conductance in only a subset of cells.

To test the third prediction we examined the effect of the CaMKII blocker on the histogram of event amplitudes in the largely affected cells, to determine if it could be computationally fully reversed by a two-fold multiplication of the amplitude of each event. For each cell in the affected cluster the fraction of the events affected by the CaMKII blockers was calculated (Materials and Methods) and accordingly a randomly chosen set of events was multiplied by a factor of two. A new amplitude distribution curve was calculated from the whole set of data, both the multiplied and the non-multiplied events. This manipulation completely reversed the blockers' effect on the event amplitude distribution curve (Fig 1C and 1G; KN93: P<0.001; tatCN21: P< 0.01; where the P value is the likelihood that the two distribution curves are drawn from different populations). Remarkably, the reverse of the blocker effect was evident not only in the average amplitude distribution curve but also when examining each cell separately, yielding a P value of 0.004±0.0012 for KN93 and a P value of 0.006±0.0017 for tatCN21 (Fig 1D and 1H).

**Fig 2:**
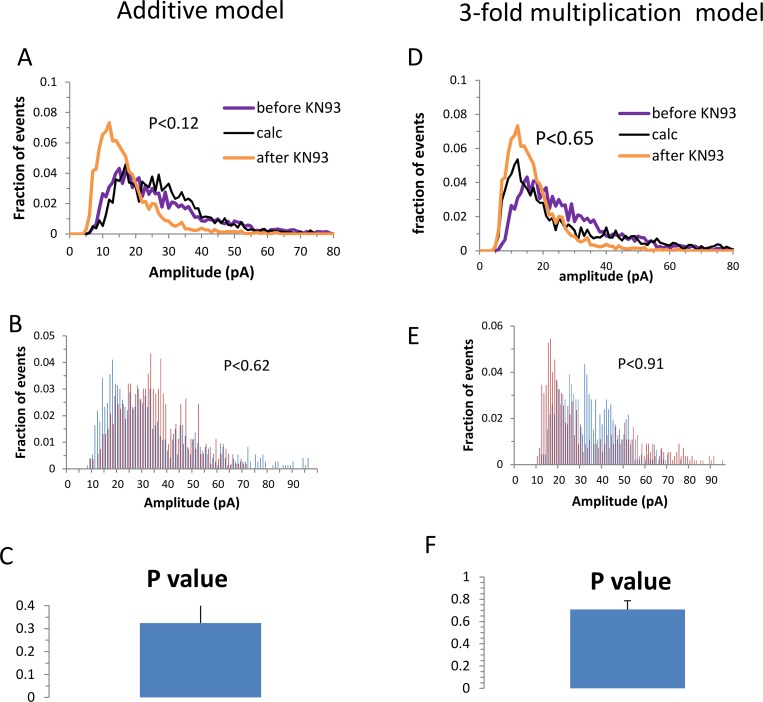
The CaMKII blocker effect cannot be reversed by an additive model (left) or a 3-fold multiplicative model (right). **A, D:** Amplitude distribution curve before and after CaMKII blocker (as in Fig 1C). The black curve in A was calculated assuming a model in which the amplitude of each event was increased by a random number ranging from zero to twice the difference between the average event amplitude before the blocker and after the blocker. The black curve in D was calculated assuming a model of multiplication by a factor of 3, where the fraction of events that were affected by the blocker was calculated for each cell and the calculated amplitude distribution curve was reconstructed accordingly. In both models the reverse calculation assuming these models did not yield an effective reversal of the blocker effect on the amplitudes distribution curve. **B, E** The effect of the blocker could not be reversed on a single cell basis, yielding a high average P value as was shown in the **C, F.**

Does our ability to computationally reverse the effect of CaMKII blocker on the amplitude distribution curve indeed validate our assumed model of two-fold multiplication? We examined the sensitivity of the amplitude distribution curve for the hypothesized model and attempted to reverse the blocker effect using two models that were different from our model. In the first model (Fig 2, left panel) we assumed that for each synapse a random number of GABA_A_ channels were added. In the second model (Fig 2 right panel) we assumed that the amplitude of all events was multiplied by a factor of three. For both models, although the average event amplitude was restored to the level before the blocker (a ratio of 0.99±0.01 and 1.00±0.02), the effect of the blocker on the average distribution curve could not be reversed (P<0.12 and P<0.65 for the average distribution, Fig 2A and 2D; for the single cells P<0.32±0.21 and P<0.7±0.17 Fig 2B, 2C, 2E and 2F).

**Fig 3:**
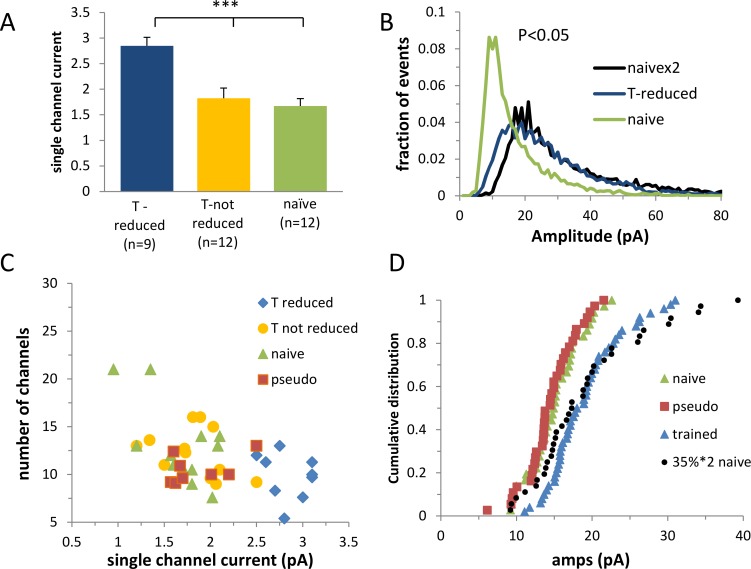
The cells in the CaMKII blocker affected cluster were affected by the task learning through whole-cell twofold multiplication of the GABA_A_ single-channel current. **A.** The average single channel current of the cells in the CaMKII affected cluster from the trained group (T-reduced) was approximately twice that of the average single channel current in the naïve group and the non affected trained group (T–not reduced). **B.** The amplitude distribution curve of the cells in the affected cluster in the trained group could be reconstructed by multiplying all events in the naive group by a factor of two (T-reduced, n = 9; T-not reduced, n = 12; naïve, n = 12). **C.** The trained group deviated from the control group mainly by the presence of the sub-group of cells that exhibited a large single channel current. **D.** The cumulative frequency distribution of the trained group could be explained by multiplying by two the average event amplitude of a randomly selected 35% of the cells in the naïve group (P<0.34 Kolmogorov-Smirnov test). Each point represents the average event amplitude in a neuron (x-axis).

Next we tested the fourth prediction and examined whether the cells that were largely affected by CaMKII were likely to be the cells that were modified by the task learning in the manner predicted by the memory amplification model. We compared the cells in the affected group before CaMKII blocker was applied to the cells in the control groups. According to the amplification model, their single channel current was expected to be twofold higher than the single channel current of the controls, and their amplitude distribution curve should be reconstructed by multiplying all the events in the control group by a factor of two. We found that the single channel current averaged over all the largely affected cells, both from the KN93 and from tatCN21 experiments, was almost twofold higher than the average single channel current in the naïve group and in the non-affected cells from the trained group (Fig 3A). Moreover, the amplitude distribution curve averaged over the largely affected cells prior to the CaMKII blocker application could be reconstructed by doubling the amplitude of all events from the naïve group (P<0.05, Fig 3B).

If the diverged effect of the CaMKII blocker on the cells recorded from trained animals is a consequence of the diverged effect of learning the task, we expect that the affected subset of neurons would deviate from the non-affected neurons prior to the CaMKII blocker application. Indeed, the same cells that were selected based on the large effect of CaMKII blocker could be clearly segregated based on their single channel current, but not on their number of GABA_A_ channels (P<0.2) as predicted by the memory amplification model (Fig 3C).

We concluded that all model predictions were satisfied and therefore that learning the task induces a whole-cell, two-fold increase of GABA_A_ synaptic receptors in a subset of cells.

Next we inquired whether in addition to the changes required by the memory amplification mechanism, learning had induced an additional measurable modification of the synaptic transmission. We approached this by examining if the distribution of averaged event amplitude of cells from the trained group can be reconstructed using cells from the naïve group assuming the memory amplification mechanism: The averaged event amplitude of a randomly selected subset of neurons from the naïve group was multiplied by two. The subset of cells entailed 35% of the neurons which is the proportion of largely affected cells in this data set. We found that the calculated cumulative frequency curve of the cell’s averaged event amplitude was similar (P<0.34 Kolmogorov-Smirnov test) to the experimental cumulative frequency curve of the trained group (Fig 3D), suggesting that the memory amplification model can account for virtually all task learning induced modifications in synaptic strength.

#### Effect on AMPA mediated events

The memory amplification model requires the modulation of the GABA_A_ synapses to be paralleled by the same modulation of the AMPA mediated synapses, such that the four predictions regarding the effect on the GABA_A_ mediated events can also be confirmed for the AMPA mediated events. Since CaMKII is known to double the AMPA channel conductance we expected that CaMKII blocker would have the same effect on the AMPA mediated events as it had on the GABA_A_ mediated events.

**Fig 4:**
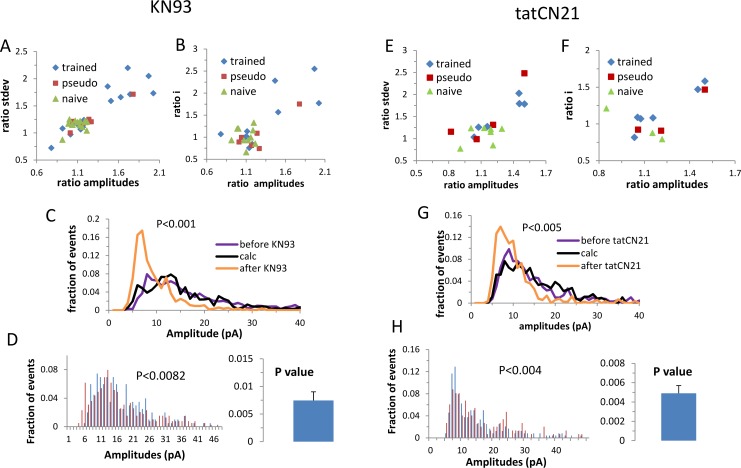
CaMKII blockers (KN93 left; tatCN21 right) act through a whole-cell twofold division of the AMPA single channel conductance in a subset of cells from trained rats. **A, E For each cell, the effect of CaMKII blocker on the average and standard deviation of the AMPA mediated event amplitudes is shown. The effect of the CaMKII blocker exhibits a clear division into two clusters (KN93: trained, n = 16; pseudo, n = 6; naïve, n = 14. tatCN21: trained, n = 7; pseudo, n = 5; naïve, n = 7). B, F** Similarly, the effect of CaMKII blocker on the AMPA mediated average event amplitude and AMPAR single channel current exhibits two clusters (KN93: trained, n = 10; pseudo, n = 6; naïve, n = 10. tatCN21: trained, n = 6; pseudo, n = 3; naïve, n = 4). **C, G** A distribution curve of the AMPA mediated event amplitude averaged for all cells in the affected cluster recorded before and after the CaMKII blocker (KN93: n = 5; tatCN21: n = 4). The black curve computes the reverse of the blocker effect under the multiplication model after calculating for each cell the fraction of events that were affected by the blocker (see Fig 1C and 1G). **D, H** the blocker effect could be reversed for each cell separately. Left: an example of one cell; right: the P value was calculated for each cell and the average of these P values was calculated from all cells in the affected cluster.

Similar to GABA_A,_ the first prediction was examined by analyzing for each cell the effect of the CaMKII blocker on the average and standard deviation of the event amplitudes. We found that both CaMKII blockers (tatCN21, KN93) had a clustered effect (mean silhouette values: KN93 0.75; tatCN21: 0.72) on the cells (Fig 4A and 4E), where the affected cluster exhibited a large CaMKII blocker effect (the averaged event amplitudes before applying the blocker divided by the average event amplitudes after; KN93: 173±21%, tat: 148±3%) and the non-affected cluster exhibited a small effect (kn93:105±11%, tatCN21: 109±12%). While only a small fraction of the cells from the control groups was in the affected cluster (kn93: 5%, tatCN21: 7%), neither CaMKII blockers had a homogenous effect on the trained group, where 44% of the kn93 trained cells and 43% of the tatCN21 trained cells clearly differed from the rest of the trained group and the control groups. Here too, the clustered effect of the CaMKII blockers does not reflect animal-dependent variability since for almost every cell in the affected cluster there was another cell from the same animal that belonged to the less affected cluster (6 out of 7 for KN93, and 2 out of 3 for the tatCN21).

As stated in the second prediction, CaMKII blockers had a clustered effect as well on the single channel current (mean silhouette values: KN93 0.74; tatCN21 0.74), where the cells that exhibited a pronounced reduction in the average event amplitude exhibited a similarly pronounced reduction in the AMPA single channel current (KN93 173±22%, tatCN21 148±6%; Fig 4B and 4F). Similar to inhibition, the effect of the blockers was partial, where KN93 had a significantly larger affect than tatCN21. Calculating the fraction of events that were affected by the blocker (see Materials and Methods) in the affected cells yielded that 73% of the events were affected by KN93 blocker and 48% of the events were affected by tatCN21. Similar to inhibition we tested the third prediction by examining if for each cell in the affected cluster the effect of the CaMKII blocker could be fully computationally reversed by multiplying the events’ amplitudes by a factor of two, after calculating the fraction of affected events for this cell. Remarkably, a complete reversal was observed both when averaged over all the affected cells (Fig 4C and 4G) and for each cell separately (Fig 4D and 4H).

**Fig 5:**
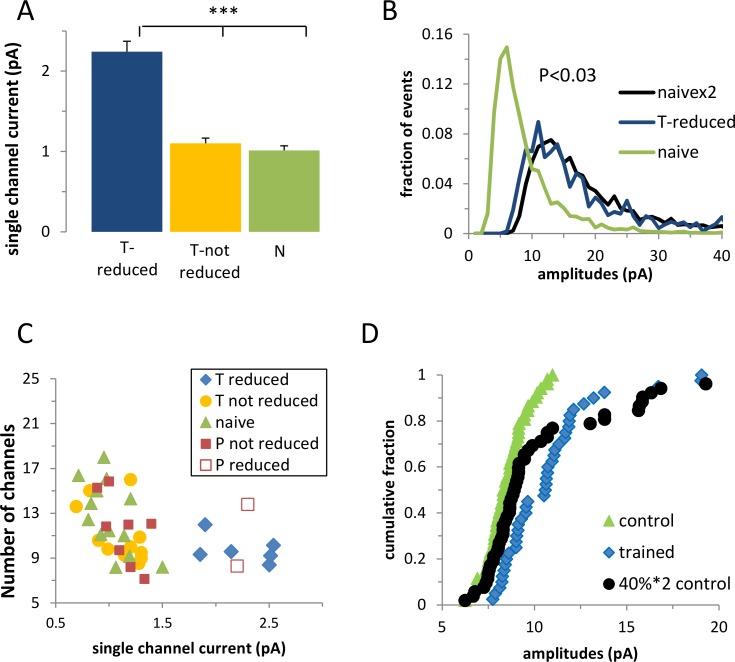
The cells in the CaMKII blocker affected cluster were affected by the task learning through whole-cell twofold multiplication of the AMPA single-channel current. A. The average single channel current of the cells in the CaMKII affected cluster from the trained group (T-reduced, n = 6) was approximately twice that of the average single channel current in the naive group (n = 13) and the non-affected cells in the trained group (T-not reduced, n = 11). B. The amplitude distribution curve of the cells in the more affected cluster could be reconstructed by multiplying all events in the naive group by a factor of two (T-reduced, n = 6; T-not reduced, n = 11; naïve, n = 13). C. The trained group deviated from the control group mainly by the sub-group of cells that exhibited a high single channel current. D. The cumulative frequency curve of the cell’s average distribution curve could not be fully explained by two-fold multiplication of the average event amplitude of a randomly selected 40% of the cells in the naive group.

We tested the fourth prediction by examining whether the neurons that were affected by the CaMKII blocker were also the neurons that were modified by learning the task according to the memory amplification model. These cells exhibited an average single channel current that was twofold higher than in the controls and the non-affected trained cells (Fig 5A). Moreover, their amplitude distribution curve could be constructed by doubling the amplitude of all events in the control group (Fig 5B). Indeed, these cells could be segregated from the non-affected cells based on their AMPA single channel current, but not based on the number of channels (P<0.15; Fig 5C). We concluded that also for the AMPA mediated synaptic transmission all model predictions were satisfied.

Next we examined whether the memory amplification model accounts for all learning induced effects on AMPA mediated synaptic transmission. The averaged event amplitude of a randomly selected 40% of the cells in the naive group was multiplied by two. While we could reproduce the observed break in the cumulative frequency curve fairly well (Fig 5D), we could not reproduce the shift (P<0.01 Kolmogorov-Smirnov test). Therefore, it is likely that learning the task induced an additional process that modulated the excitatory event amplitude homogenously in all cells.

In summary, our experimental data confirmed the four predictions for both inhibition and excitation, and therefore support the whole-cell amplification model.

### Modeling

In this part, we aim to explore the functional significance of whole-cell synaptic multiplication, in which the strength of all excitatory and all inhibitory synapses in a single cell was doubled. We previously showed in a preliminary study, using integrate and fire neurons, that such a whole-cell two-fold multiplication in synaptic strength functions as a memory amplification mechanism [9]. Here we attempt to bridge the gap between the simplified integrate and fire neuron that was used in the network simulations and a more realistic biophysical neuron. To that end, using biophysical simulations, we first characterize the effect of a whole-cell, two-fold increase in synaptic strength on the neurons' firing response. A detailed characterization of the effect of the whole-cell amplification mechanism on the number of spikes should enable a better understanding of its effect on memory amplification. An amplifier whose main effect on the single cell firing response is at the near-threshold level should have a different effect on memory amplification than an amplifier whose main effect is at the supra-threshold level. Moreover, an amplifier which instantaneously follows the changes in synaptic input is likely to exert a different effect than a history dependent amplifier.

We used a single cell with a simplified morphology and realistic biophysical properties (see Materials and Methods), where realistic post-synaptic excitatory and inhibitory responses were implemented along the basal and apical dendritic cylinders. In order to simulate synaptic bombardment as recorded in vivo we mimicked the firing of populations of inhibitory and excitatory pre-synaptic neurons using an independent random Poisson process such that the reversal potential of the net synaptic current (see Material and Methods) during background activity was about -37 mV [20].

#### The amplification mechanism scales the net synaptic current without affecting its polarity

**Fig 6:**
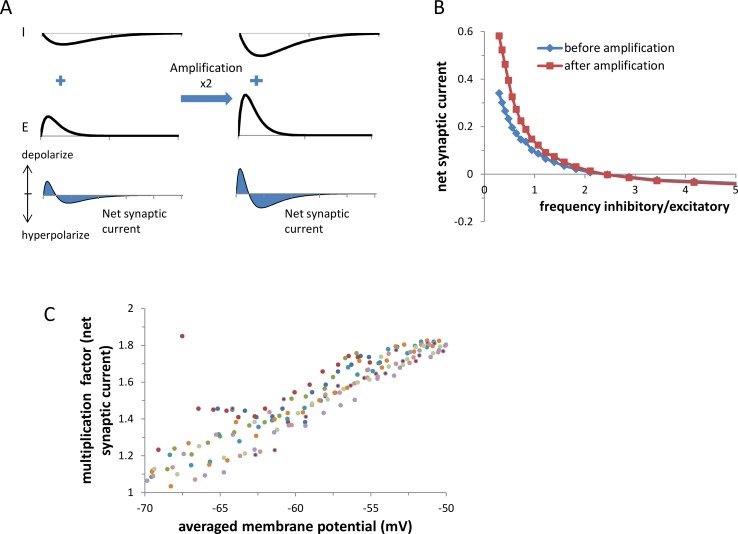
The amplification mechanism multiplied the net synaptic current. **A.** The net synaptic current is the sum of the inhibitory and excitatory currents. Multiplication of both the inhibitory (upper panel) and excitatory currents (middle panel) multiplies the net synaptic current (lower panel). Therefore, if the net synaptic current is positive this multiplication will further depolarize the cell and if negative will further hyperpolarize the cell. **B.** The ratio of the activation frequency of inhibitory to excitatory synapses was varied by decreasing the activation frequency of each inhibitory synapse from11Hz to 3Hz, and increasing the activation frequency of each excitatory synapses from2Hz to 10Hz by 0.5Hz (x-axis). Each point on the graph is the average of 10 simulations. The different ratios of activation frequencies (inhibitory activation frequency divided by the excitatory activation frequency x-axis) resulted in different net synaptic currents (y-axis). The amplification mechanism increased the net-synaptic current while maintaining its polarity. A significant multiplicative effect was mainly observed at positive net synaptic currents, thus primarily when the effect of the net synaptic current was excitatory. This figure is a result of one simulated cell. **C.** Multiplication factors of the net synaptic current as a function of the average membrane potential. The simulation protocol is as described in B, where each color indicates a different set of simulations (in total 10 different sets of simulations). The sets of simulations differed in terms of intrinsic characteristics (resting potential: -90 - -60 mV) and the average strength of the synapses (excit/inhib: 0.6–1.3). Each dot in a set of simulations is a result of a different activation frequency of the inhibitory and excitatory synapses, as was described in B.

The net synaptic current at each moment is defined as I_inhib_(t)+I_excit_(t) and is the sum of the currents mediated by all active inhibitory and all active excitatory synapses at time t. When the inhibitory synaptic current overcomes the excitatory current, the net synaptic current is negative and will hyperpolarize the membrane potential (Fig 6A). When the excitatory current is stronger, the net synaptic current is positive and will depolarize the membrane potential. Multiplication of both the excitatory and inhibitory currents by the amplification mechanism [9] will multiply the sum of the excitatory and the inhibitory currents at any given time and will therefore multiply the net synaptic current (Fig 6A). Here, we further tested whether such multiplication would hold at a more realistic biophysical cell in order to include the possible additional effects of reversal potential and voltage dependent currents on the net synaptic current.

We first examined whether the synaptic multiplication maintains the polarity of the net synaptic current. These properties can enable the amplification mechanism to act as a precise amplifier that amplifies the cell’s response without affecting its information. We conducted a set of simulations in which the size and the polarity of the net synaptic current were varied by simultaneously decreasing the activation frequency of each inhibitory synapse and increasing the activation frequency of each excitatory synapse.

Multiplication of both excitatory and inhibitory conductance by whole-cell balanced amplification scaled the net synaptic current, with no effect on its reversal point (Fig 6B). At a positive net synaptic current, the amplification mechanism had a larger effect due to the larger driving force of the excitatory synapses as compared to the inhibitory synapses. The multiplication factor of the net synaptic current was small when the average membrane potential approached the GABA_A_ reversal potential and became higher and more stable (1.4–1.8) as the average membrane potential became more depolarized (Fig 6C). Thus, the effect of the amplification mechanism on the net synaptic current was correlated with its initial size and its polarity, indicating that the inhibition-excitation balance was maintained as well after its induction. Moreover, the amplification of the net synaptic current tended to increase as the neuron received a larger proportion of excitatory synaptic input.

#### Whole-cell balanced amplification increases the gain of the spiking response

The magnitude of the excitatory synaptic current can be modified by increasing the relative contribution of the strong excitatory synapses, or by homogeneously increasing the activation of all excitatory synapses (Materials and Methods).

We first examined the effect of the amplification mechanism in simulations where the net synaptic input was varied by increasing the activation frequency of strong excitatory synapses and decreasing the activation frequency of the weak excitatory synapses (see Material and Methods). Such increase in the proportion of activated strong synapses is expected when the cell participates in an active learned memory pattern.

**Fig 7:**
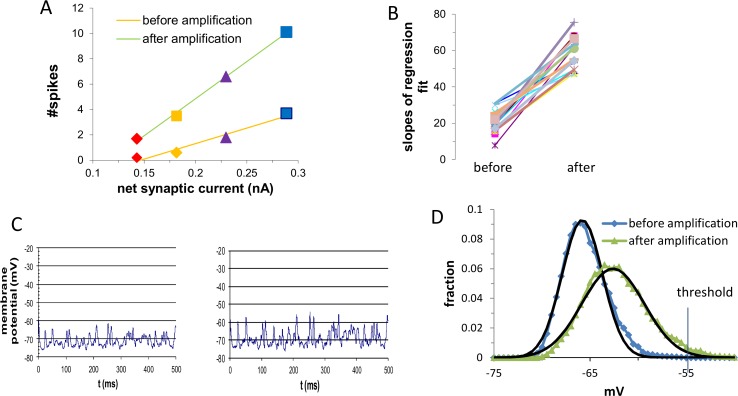
Whole-cell balanced amplification increased the slope of the input-output curve through an increase of the average and the stdev of the membrane potential. **A.** The number of spikes measured during an interval of 500 ms, before (yellow) and after (green) whole-cell balanced amplification is shown for increasing fractions of active strong synapses (red: 15%; orange: 30%; purple: 45% and blue: 60%) as evidenced by the increased net synaptic current (X-axis). Whole cell balanced amplification increased the gain of the spiking response to the same input. **B.** Slopes of the regression lines before (left) and after (right) whole-cell balanced amplification. In all simulations (20 different sets of simulations) the data matched a linear fit (r>0.95) **C.** Membrane potential before (left) and after (right) the amplification mechanism was applied.**D.** Distribution of the membrane potential before and after the amplification mechanism was applied. Whole-cell balanced amplification increased both the average and the standard deviation of the membrane potential. Black lines indicates the fit to a normal distribution where the amplification shifted the average by 3.7 mV and multiplied the stdev by 1.6.

We used the size of the net synaptic current before the amplification mechanism was induced as an indicator of the strength of the total synaptic input. In Fig 7A it can be seen that increasing the relative contribution of strong synapses increased the net synaptic current, which elicited a linear increase in the number of spikes. As shown in this example, the amplification mechanism increased the slope of the input-output curve and as such increased the spiking response in a multiplicative fashion; namely, the gain of the cell’s response to the same synaptic activity pattern increased. In Fig 7B, for each point in the parameter space (i.e. for each simulated cell) the slope of the regression line after the induction of the amplification mechanism differed from the regression in the same cell prior to the amplification. In all the simulations whole-cell balanced amplification increased the slopes significantly (P<0.0001) by a factor that ranged from 1.6 to 4.8 with an average of 2.75±1.5. We concluded that the amplification mechanism effectively increased the gain of the cell for the same input, and as such may be a mechanism for gain modulation. Gain modulation is a change in the slope of the firing rate curve corresponding to a multiplicative scaling which is distinct from an additive shift. The multiplicative effect on the number of spikes of the amplification mechanism supports our previous hypothesis of memory amplification. Thus, when the cell is not tuned to the synaptic input and thus receives a small net synaptic current, whole-cell amplification appears to induce a small net effect; however, when the cell participates in an active memory-pattern and thus receives a large excitatory net synaptic input, the net effect of whole cell amplification is likely to be large.

The multiplicative effect of the amplification mechanism on the number of spikes can be attributed to its multiplicative effect on the net synaptic current, where for all activation strengths and over all simulations, the average amplification of the net synaptic current was similar and amounted to a factor of 1.47±0.06 (a range of 1.42 1.62). Such a relatively uniform scaling factor demonstrates the stability and the robustness of whole-cell balanced amplification. The multiplication factor of the net synaptic current was considerably less than the multiplication factor of the inhibitory and excitatory conductance’s (2.0) due to the increased average membrane potential following the amplification mechanism (from an average of -66.6 mV to an average of -64.5 mV) which specifically increased the inhibition driving force.

We next examined whether the increase in number of spikes was also mediated by the increase in variance of the net synaptic current as a result of the amplification mechanism.

#### The effect of whole-cell balanced amplification on spiking response is also mediated via its effect on the fluctuations of the membrane potential

Whole-cell balanced amplification multiplied the standard deviation of the net synaptic current (Fig 7C) by a value that was similar to the increase in its mean. This is a direct consequence of the multiplication of the strength of the inhibitory and excitatory conductance. For all simulations, the average multiplication factor of the standard deviation of the net synaptic current was similar and amounted to a value of 1.70±0.07 (a range of 1.53–1.79).

The increase in standard deviation as a result of the amplification mechanism can be derived from the equation below. This equation calculates an approximation of the variance of a synaptic current that results from **N** synaptic events with decay time constant *τ*, average EPSC amplitude $\bar{\omega }$, coefficient variation of amplitudes **CV** in a time interval **T** assuming that *τ* ≪ *T* (see Appendix 1 in S1 Text):

$$\sigma^{2}={\bar{\omega }}^{2}\cdot N\cdot \frac{\tau }{T}\cdot \left(\frac{1}{2}+\frac{\tau }{T}\cdot \left(CV^{2}-1\right)\right),$$ (1)

The variance has a quadratic dependency on the average amplitude of the synaptic event. Thus, it is consistent with our results that multiplying the current mediated by all synaptic sites through the amplification mechanism has the same effect on the fluctuation of the net synaptic current as its effect on its average. It has been shown both experimentally and theoretically that increasing the variance of the membrane potential while preserving its mean increases the number of spikes [21–22]. Thus whole-cell balanced amplification increases the number of spikes not only through the increase in the mean net synaptic current but also through the increase in its variance. The effect of the amplification mechanism is illustrated in Fig 7D, where we calculated the voltage distribution before and after the amplification. The amplification mechanism affected both the average and the standard deviation of the membrane potential. This resulted in a higher proportion of the membrane potential that exceeded threshold and consequently, amplified the spiking response of the cell.

In Eq 1 it can be seen that increasing the average amplitude of a synaptic response ($\bar{\omega }$) has a much stronger effect on the variance than increasing the number of active synapses (N). Next we examined the effect of whole-cell balanced amplification on spiking activity when the increase in excitatory synaptic input resulted from a homogenous increase in activation frequency of the excitatory synapses rather than an increase in the relative contribution of strong synapses. For all activity strengths, the activation frequency was increased such that the increase in net synaptic current was identical to the increase found in previous simulations (a ratio of 0.998±0.011). In agreement with the analytical results we found that increasing the proportion of strong synapses resulted in an increase of 25% in the standard deviation, whereas increasing the synaptic activation frequency only resulted in an increase of 5%. This difference was reflected in the slope of the input-output curve, which was 32% higher when induced by increased proportions of strong synapses. Despite these differences between the two sets of simulations, the amplification mechanism multiplied the slope of the input-output curve by a similar factor (P<0.65) demonstrating the stability and the robustness of this amplification mechanism.

#### Whole-cell amplification functions as a linear multiplicative amplifier in the balanced state

A significant body of theoretical and experimental evidence suggests that the neuronal network exists in the regime of a balanced state, where excitatory and inhibitory synaptic inputs balance the mean values of the synaptic current but add their fluctuations [23].The network in the balanced state is capable of fast tracking the temporal changes in the external input to the network. The mode of activity in this state is referred to as the “asynchronous state” or “rate mode”.

Whole cell balanced amplification multiplies the strength of all inhibitory and excitatory synapses equally and therefore maintains the balanced state. Our findings, which demonstrate that the average and the standard deviation of the net synaptic current are multiplied by almost the same factor, maintain the outcome of the balanced state in which the network’s average net synaptic current is much smaller than its fluctuations. One of the characteristics of the balanced state is that the synaptic inputs are very weakly correlated. As a consequence, the fluctuations of the net synaptic current obey Gaussian statistics characterized by the variance and the mean of the net synaptic current. Indeed, the voltage distribution both before and after balanced amplification obeyed the normal distribution (r^2^ = 0.98, Fig 7D) and the effect on the standard deviation and the average, fit the values that we reported in previous sections (ratio of stdev: 1.65±0.09 mV; increase in average potential: 3±0.7 mV).

Since whole-cell balanced amplification is independent of past activity, we could derive its impact on the number of spikes from its impact on the voltage distribution. Assuming a linear threshold neuron in which below threshold the firing frequency is zero and above threshold it is a linear function of the net synaptic current, the firing frequency can be evaluated through a convolution of the Gaussian function and the linear threshold transfer function of the neuron, resulting in the following equation (see Appendix 2 in S2 Text):

$$F\left(\bar{I},\sigma \right)=\beta \cdot \sigma \left(\frac{1}{\sqrt{\pi }}\cdot e^{-{\left(\frac{\bar{I}-\theta }{\sigma \sqrt{2}}\right)}^{2}}+\frac{\left(\bar{I}-\theta \right)}{2\sigma }\cdot \left(1+\text{erf}\left(\frac{\bar{I}-\theta }{\sigma \sqrt{2}}\right)\right)\right)$$ (2)

where *θ* is the net synaptic current that is required to reach threshold, σ is the standard deviation of the synaptic current, $\bar{I}$ is the mean of the net synaptic current, and β is the multiplication factor of the linear transfer function. Using this equation, whole- cell balanced amplification can be induced by multiplying σ and $\bar{I}$ by the same factor (which as a result of the simulation was determined to be 1.7). Thus, the amplification factor can be estimated from Eq 2 by calculating the ratio between the number of spikes before and after whole-cell balanced amplification. Under this model, the amplification factor is independent of the exact implementation of the input-output relation (β) and the whole parameter space can be covered solely by varying the terms $\frac{\theta }{\sigma }$ and $\frac{\bar{I}}{\sigma }$.

**Fig 8:**
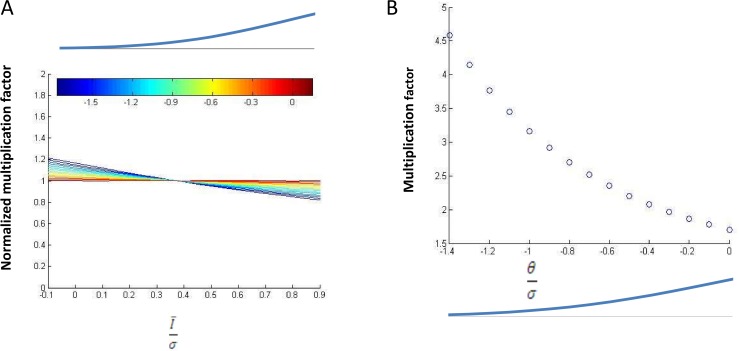
In the balanced state, whole cell balanced amplification functions as a stable and robust linear amplifier. **A.** For each level of intrinsic excitability (indicated by the color bar) the multiplication factor induced by whole-cell balanced amplification was calculated for different strengths of net synaptic input ($\frac{\bar{I}}{\sigma }$, x-axis) using Eq 2. We examined the sensitivity of the multiplication factor to the net synaptic current by normalizing it to its mean value (Y -axis). For all intrinsic excitability levels ($\frac{\theta }{\sigma }$), indicated by the color bar), the value was close to one, indicating an almost constant multiplication factor. The image above the color bar helps to visualize $\frac{\theta }{\sigma }$, by indicating for each value of $\frac{\theta }{\sigma }$ (and thus for the aligned color) the proportion of the voltage above the threshold when the mean synaptic current was zero. **B.** The mean multiplication factor (Y-axis) was calculated for each level of intrinsic excitability ($\frac{\theta }{\sigma }$, X-axis).

Whereas the size of fluctuations was suggested to be mainly determined by the network activity [19], the spiking threshold and the size of the average net synaptic current were suggested to differ between neurons as a result of non-homogenous intrinsic excitability, random synaptic connectivity and non-homogenous external input. To account for the variability that result from differences in intrinsic variability, we examined the amplification factor induced by whole-cell balanced amplification at different values of spiking threshold $\left(\frac{\theta }{\sigma }\right)$. For each value of intrinsic excitability $\left(\frac{\theta }{\sigma }\right)$, we varied the mean net synaptic input ($\frac{\bar{I}}{\sigma }$) such that $-0.1<\frac{\bar{I}}{\sigma }<0.9$, yielding a range of synaptic inputs in which the average net synaptic current was smaller than its fluctuation. We found that for a large range, the multiplication factor induced by whole cell balanced amplification was nearly independent of the magnitude of the mean net synaptic input (Fig 8A). As the neuron’s intrinsic excitability shifted further from threshold $\left(\frac{\theta }{\sigma }>-0.8\right)$, the amplification factor exhibited a mild dependency on the net synaptic current as expected near threshold. This result solidifies our observation from simulations that the amplification mechanism functions as a linear amplifier on the number of spikes.

Next we investigated the sensitivity of the multiplication factor induced by whole-cell balanced amplification on the intrinsic excitability of the neuron. Although $\left(\frac{\theta }{\sigma }\right)$ varied considerably, the variation in the amplification factor was small (1.7–3.5). At low intrinsic excitability the amplification factor was higher, as is expected when the neuron is in the threshold range.

We showed that in the balanced state regime, whole cell balanced amplification functions as a linear amplifier, where its multiplication factor is stable and exhibits low variability at different states. The range of amplification factors that were found analytically are similar to the amplification factors found in the simulations. This similarity between the analytical study and the simulation study indicates that these results are resistant to the specifics of implementation and characteristics of the neuron’s physiology.

#### Whole-cell amplification functions as an instantaneous amplifier

So far, the impact of whole-cell balanced amplification was studied in conditions where the mean membrane potential was constant and where Poisson-like fluctuations in the membrane potential cause irregular spiking activity. Next we examined the effect induced by the amplification mechanism when the mean membrane potential is briefly elevated due to an orchestrated increase of the synaptic input that is considerably higher than the ongoing fluctuations induced by synaptic noise [24]. In-vivo, such brief and orchestrated elevations of the membrane potentials can be a result of a brief external stimulus such as touch, sound or visual stimulation [25]. A reliable spiking response during these voltage bumps enables the neuron to reliably transfer the information conveyed by these bumps to other neurons. However the irregularity of the voltage due to synaptic noise reduces the ability of the neuron to reliably elicit a spike during these voltage excursions.

**Fig 9:**
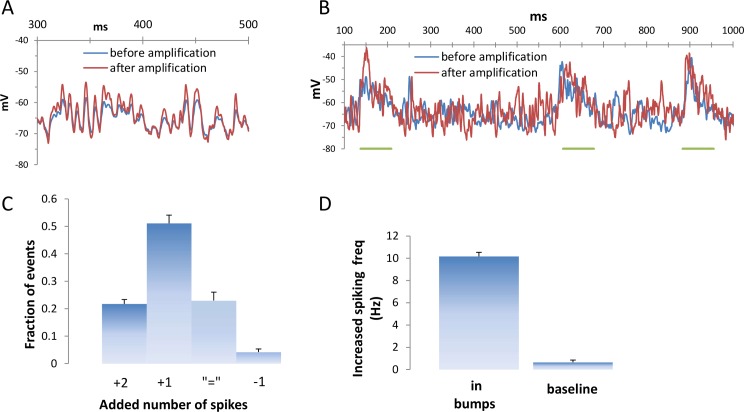
Whole-cell amplification functions as an instantaneous amplifier. **A.** At the peaks of the fluctuations the effect of the amplification was larger. The synaptic activity exhibited the same pattern before and after the amplification thus demonstrating the instantaneous effect of whole-cell balanced amplification. **B.** The probability of the excitatory synapses was increased according to a time course described by an alpha function (tau = 24 ms), with a multiplication factor of 5, resulting in voltage transients that were considerably larger than the ongoing voltage fluctuations with a half time around 90 ms. Despite a different synaptic activity pattern, and the relatively short duration of these transients the amplification induced by whole-cell balanced amplification was very evident. In both traces the sodium conductance was set to zero to eliminate the action potentials. **C.** Whole-cell balanced amplification induced robust event based amplification where in the large majority of the synchronized events (74%) the number of spikes increased. Simulation were performed at 30 different simulation sets at which both resting potential and synaptic strength were varied (see Materials and Methods) **D.** Due to the multiplicative nature of whole-cell amplification, the increase in the number of spikes was pronounced during the increased voltage transients while the increase in the number of spikes during the baseline was negligible.

The continuous multiplication of the net synaptic current by the amplification mechanism and its independence of previous spike activity enable it to respond instantaneously to changes in the membrane potential, where its effect is especially pronounced at the peaks of the depolarized fluctuations (Fig 9A). Thus we expected that the amplification would reliably increase the number of spikes that these bumps elicit, despite differences in ongoing synaptic activity (Fig 9B). To test the consistency of the impact of amplification in these conditions we conducted a set of simulations with ongoing varying synaptic noise, where the elevated synaptic activity was randomly located in time.

First we investigated the impact of the amplification mechanism on the number of spikes during a brief (90 ms) elevation of synaptic activity. Despite the ongoing varying synaptic noise (Fig 9B), the amplification increased the number of spikes in the large majority of the bumps (74%, Fig 9C). The number of spikes did not differ for 23% of the bumps, and had decreased in less than 5% of the bumps. Thus the amplification mechanism appeared to reliably amplify the number of spikes during almost every event of elevated excitatory synaptic activity, despite the ongoing variability in synaptic noise. Due to the multiplicative effect of whole-cell amplification its net effect was pronounced primarily during the periods of elevated activity: while the increase in average firing frequency as a result of the amplification mechanism was amounted to 10.1±0.3 Hz during the bumps, during baseline the increase amounted only to 0.65±0.2 Hz (Fig 9D). The multiplication factors of the number of spikes during these bumps were similar to the ones reported in the previous sections.

**Fig 10:**
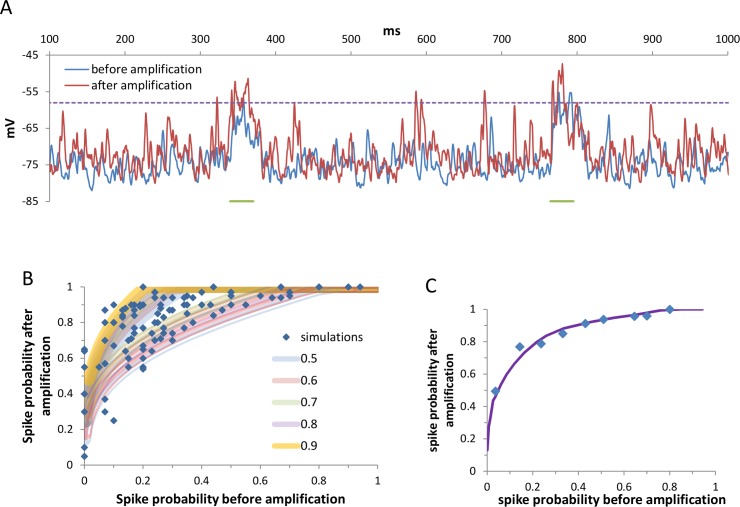
Whole-cell balanced amplification had a considerable impact on the probability that very short voltage transients would elicit a spike. **A.** An alpha function-like time course of increased rate activation of excitatory synapses was induced (tau = 12 ms, multiplication factor = 5) resulting in brief (half time ~ 30 ms) excursions of voltage bumps. **B.** For each trace (30 bumps of voltage elevations) the probability of eliciting a spike was calculated before and after whole cell balanced amplification. The simulation parameter space was spanned by varying the resting membrane potential, the synaptic strength, and the activation frequency of the synapses as indicated in the Materials and Methods. Binning the probabilities in all conditions resulted in highly non-linear amplification, where bumps that had a low likelihood of eliciting a spike were amplified to threshold values with probabilities of 50%. Using Eq 2 we tested whether the multiplication of σ and $\bar{I}$ by a factor of 1.7 could account for the simulation results. β was set such that the maximum frequency was 100 Hz or 150 Hz [58] at values where the average current was 3σ more depolarized than threshold (a range in which sodium channels still do not undergo pronounced inactivation). The parameter space (35 different sets of simulations) was spanned by varying $\frac{\bar{I}}{\sigma }$ (0.4–0.9, similar to the values obtained in the simulations) and the threshold $\frac{\theta }{\sigma }$ (0.5–3.7), yielding results that spanned the same area as the simulations. The values of $\frac{\bar{I}}{\sigma }$ are indicated by color coding, and the values of β are indicated by solid lines (max firing frequency of 150 Hz) and double lines (max firing frequency of 100 Hz). **C.** Binning these calculated values based on the probability before whole-cell amplification yielded a curve that was similar to the simulated curve.

Next we examined the impact of the amplification mechanism on the probability of eliciting a spike during very brief (30 ms) periods of elevated activity (Fig 10A). The parameter space was explored by modulating the resting membrane potential, and hence the threshold distance, and by modulating the strength of the excitatory and inhibitory synaptic responses. Despite the brief interval and the irregularity of both the synaptic noise and the bumps, the effect of the amplification mechanism on the probability of eliciting a spike was striking (Fig 10B and 10C). Even at probabilities just above zero, whole-cell amplification increased the firing probability of the cell to threshold values, and thus to approximately 50% chance. When the cell was near the threshold value (P ≈50%), the amplification caused the cell to reliably respond to the bumps (>90%).

Finally, we examined whether the sharp increase in the probability of firing a spike during these bumps could be explained solely by multiplying the standard deviation and the average of the net synaptic current, as we observed for the effect on the firing frequency. The parameter space was spanned by varying the average net synaptic current $\left(\frac{\bar{I}}{\sigma }\right)$ and the threshold $\left(\frac{\theta }{\sigma }\right)$. The probability of eliciting spike was calculated before and after multiplication of σ and $\bar{I}$ by a factor of 1.7 using Eq 2. The calculated probabilities spanned a space that was similar to the simulated probabilities (Fig 10B), where on average the calculated curve approximated the simulated curve (Fig 10C). At zero probability of eliciting a spike, the likelihood of eliciting a spike also remained negligible after whole-cell balanced amplification. Thus whole-cell balanced amplification mainly affected the bumps that were not very distant from threshold.

## Discussion

In this study we show that a subset of cells exhibited a task learning induced CaMKII dependent process in which the single channel conductance of all AMPA and GABA_A_ synaptic receptors in the cell was doubled. Subsequently we show using both analytical and numerical studies that this process functions as a linear, real-time and history- independent amplifier of the cell’s spiking response. These features enable whole-cell amplification to have robust event-based amplification, and to reliably follow the irregular and rapid changes in the cell’s synaptic input. The multiplicative nature of the amplifier ensures that the amplification will have an impact primarily when the cell’s synaptic input is largely excitatory; hence, when the cell is tuned to the input.

### All four predictions of the model were experimentally validated

In this study we verified the hypothesis in which in a subset of cells, learning the task induced a whole-cell, CaMKII dependent process, in which all the inhibitory and excitatory synapses doubled their strength through a two-fold increase in the single channel current of the AMPA and GABA_A_ receptors.

All the predictions derived from this hypothesis were confirmed using two different blockers of CaMKII for both inhibition and excitation. Validation of the first and second predictions indicates that only a subset of the cells underwent a task learning induced CaMKII dependent phosphorylation that significantly increased the synaptic single channel current and as a consequence the average event amplitude. The clustered effect could not be explained using within-animal variation since for almost every cell in the affected cluster there was a cell from the same rat that was in the non effected cluster. Moreover, the recordings were taken from identified pyramidal neurons in layer II of the piriform cortex; therefore the clustering effect could not be ascribed to different types of cells being different [7, 8].

The blocker effect was mainly apparent in cells from the trained group (30–40% of the cells), present to a lesser extent in the pseudo-trained group (0–10%) and absent in the naïve group (0%). Thus, learning the task clearly induced the amplification process, even though the recordings were made 4–5 days after training ended.

The third prediction stated that CaMKII blocker would reduce the amplitude of each event by a factor of two. This prediction required a uniform synaptic event-based effect rather than an average effect. We approached this issue by computationally reversing the effect of the CaMKII blocker on the histogram of event amplitudes through a uniform two-fold multiplication of the events amplitudes. In each cell in the affected cluster, this manipulation yielded a full computational reversal of the blocker effect, thus confirming that the CaMKII blocker reduced the amplitude of each synaptic event by a factor of two. Due to the moderate concentrations of CaMKII blockers, the blockers did not yield a full effect on the cells in the affected cluster (see results). The effect of tatCN21 (inhibition 147±7%; excitation 148±3%) was significantly smaller than the effect of KN93 (inhibition 165±9%; excitation 173±21%). The similar effect of each blocker on inhibition and excitation supports the notion that indeed the discrepancy between the expected effect of the twofold reduction and the experimentally observed effect is due to a partial effect of the blocker. For each cell, the fraction of blocker affected events was used when calculating the reversal of the blocker effect on the amplitude distribution. This reversed blocker effect cannot be attributed to the calculated fraction since when assuming different processes we did not achieve a full reversal of the blocker effect on the events amplitude histogram, even though the effect on the averaged amplitude was reversed.

The fourth prediction stated that the cells that were affected by CaMKII blocker are the cells that were affected by the task learning; thus in these cells, there was a process that caused all the synaptic AMPA and GABA_A_ receptors to multiply their single channel current by a factor of two. This prediction was confirmed by demonstrating that the single channel current in these cells was twice as high as in the controls, and that their amplitude distribution curve could be reconstructed by two-fold multiplication of all the events in the control group. The affected cells could be discriminated from the non- affected cells solely based on their single channel current, which was indicative of their difference before the CaMKII blocker was applied.

### Learning induced an additional additive increase in the averaged strength of excitatory synapses

In addition to demonstrating the task learning effect on the CaMKII blocker-affected cells, we argued that for inhibition, the multiplication process might account for virtually all the measured learning induced modifications of the synaptic events. We showed that the two-fold multiplication of the average inhibitory event amplitude in a randomly selected 35% of the cells could fully account for the learning induced modification on the cumulative frequency curve of all the cells. However we could not fully explain the learning induced modification in the cumulative frequency curve of excitatory events. Task learning induced a shift in the cumulative curve that could not be accounted for by the multiplication process in a subset of cells. Thus learning the task induced an additional process that caused an almost constant increase in the average excitatory events amplitude in all cells in the trained group.

### The applicability of memory amplification mechanism to other brain areas

Phosphorylation of the AMPA receptor by CaMKII is known to double the single channel conductance of AMPA receptors [13–15]. These findings consolidate our basic premise that CaMKII induces a two-fold increase of the AMPA receptor conductance. Although a two-fold increase in the AMPA channel conductance as a result of CaMKII dependent phosphorylation is believed to be a universal property of the AMPA receptor, currently there are no other findings that demonstrate the induction of the memory amplification mechanism in other brain regions. However since the amplification mechanism is a whole-cell mechanism and thus appears to be independent of circuit property, the memory amplification mechanism is likely to be induced also on pyramidal cells in other brain regions. Future demonstration of the induction of this mechanism in other regions can further increase its impact, as learning is known to be supported by parallel modifications in several regions.

As expected by a mechanism that is induced through whole-cell transduction mechanism, the reversal of the memory amplification mechanism using CaMKII blockers was rapid (several minutes passed between the application of the drug in the perfusion solution and the effect of CaMKII blocker). In this set of experiments the recordings were made at a fixed interval of 4–5 days after training termination [7, 8]. This interval allows us to suggest that the memory amplification mechanism comprise a long-term effect of learning, nevertheless it does not enable us to determine the time scale of the induction of this mechanism. Resolving this issue is significant for understanding the behavioral role of the amplification mechanism in different settings.

### Mechanism for gain modulation

Using simulations we have shown that whole-cell balanced amplification acts mainly via multiplicative amplification of the average and standard deviation of the net synaptic current; as such its effect is correlated with the size and the polarity of the net synaptic current. Moreover, we showed using simulations and analytical study that in the balanced state this modification leads to a linear amplification of the number of spikes with a fairly stable multiplication factor. This trait suggests that the amplification mechanism is a robust and stable mechanism for gain modulation.

Quite a few studies have attempted to describe the mechanism of gain modulation. It has been shown that tonic inhibition creates a divisive operation during physiological excitation in which the cell is under continuous bombardment of synaptic activity [26–30]. In other studies [22, 29, 31–33], it was shown that depolarizing the membrane potential in the presence of synaptic noise increases the response gain. In both mechanisms (i.e. increase in tonic inhibition and depolarization of the membrane potential), the change has an additive effect on the input-output function, and in both, the amplitude of the baseline noise determines the shift from additive to multiplicative operations [26, 31]. Indeed, theoretical studies [31, 32] have shown that in the presence of noise, the firing frequency becomes a power-law function of the membrane potential but only for a limited range of values, and that this is primarily visible at low firing frequencies. Due to this limitation, these mechanisms cannot be considered a robust driver of gain modulation at supra-threshold conditions [34].

The mechanism we suggest here is inherently multiplicative and cell selective, and hence can cause robust cell specific gain modulation. We found that both the amplitude and the standard deviation of the net synaptic current were multiplied through a whole-cell balanced multiplication by approximately the same constant for all simulations. The simulations spanned a large space of several parameters, which effectively produced different initial conditions for the inhibition excitation ratio, and a different activation curve of the cell. We confirmed these results analytically and showed that in the balanced state whole-cell multiplication induces a linear amplifier on the number of spikes. Moreover, this analysis showed that when the cell is not in the threshold regime, the multiplication factor is fairly stable.

### Whole-cell amplification functions as an instantaneous amplifier

Theoretical works suggested that gain modulation can be mediated by changes in intrinsic excitability [35,36], and more specifically through modulation of the currents that mediate the firing frequency adaptation; however using simulations these studies indicated that the induced gain is not robust at lower firing frequencies [36]. Moreover, since pyramidal cells differ in their firing adaptation properties, the resulting gain modulation varies considerably across cells.

Gain modulation mechanisms that act through modulation of the firing frequency adaptation are dependent on the past spiking activity of the cell. In the balanced regime spiking activity is inherently irregular and therefore a consistent gain modulation effect of such mechanisms can be achieved by averaging, or by long time integration of the spiking activity. Whole-cell balanced amplification modifies the synaptic input directly and as such is not dependent on past spiking activity. Moreover its underlying multiplicative process enables it to respond in particular to large deviations in the membrane potential. These characteristics enable it to act instantaneously to rapid changes in the synaptic input. We showed that whole cell balanced multiplication could reliably amplify the number of spikes in 75% of the voltage bumps that are caused by a transient, brief elevation of excitatory synaptic activity, despite ongoing differences in synaptic noise. These characteristics enable a reliable instantaneous amplification during transient periods of brief orchestrated elevations of the membrane potential that results from a brief external stimulus such as touch, sound and visual stimulation [25, 37–39].

To summarize, to our knowledge the memory amplification mechanism is currently the only candidate for a real-time amplifier that creates a high impact also at highly supra-threshold values. While a mechanism that works through depolarizing the membrane potential is real time, it exerts its main impact mainly at near threshold, and mechanism that works through firing frequency adaptation is history dependent and thus does not react instantaneously to changes in the membrane potential.

### Whole-cell amplification as a memory amplification mechanism

Amplification of a subset of cells that comprise a memory-pattern would primarily amplify the response of these cells when memory is activated. Due to the multiplicative nature, the net effect of the amplification during baseline activity is much smaller than when the cell is tuned to the input. This tendency is increased by the larger multiplication factor of the net synaptic current at membrane potentials that are considerably more depolarized than the GABA_A_ reversal potential. This enables the amplification to have a large net effect mainly when the memory-pattern is activated and the cells receive large excitatory input. In this fashion, the amplification mechanism does not modify the information carried by the memory pattern, but only amplifies it. We showed previously [9] using a simple computational model that this mechanism induces selective memory amplification. The current work explains the underlying cellular basis of this property.

A parallel increase in inhibition and excitation was previously suggested to function as a network-wide gain increase mechanism [40, 41]. This effect is mediated by the non-specific effect of neuro-modulators which increases the membrane potential of both the excitatory and inhibitory neurons [41]. While such a mechanism can affect coding by generally impacting synaptic circuits between different regions [40], the mechanism presented here is cell-specific and thus can affect coding by amplifying an input specific response.

A pattern of cells that underlies a critical behavioral response should reliably respond to the input, even during brief periods. Increasing the reliability of eliciting a spike in each of these transient periods can create a consistent response in such states. We showed that the effect of whole-cell multiplication on the probability of eliciting a spike possesses the following properties (a) at threshold values the probability of eliciting a spike was increased nearly to one and (b) the increase in probability was prominent only if the ability of the event to elicit a spike was not zero. These properties enable whole-cell multiplication to induce reliable coding without modifying the information carried by the synaptic input.

### Relation to previously described forms of synaptic plasticity

The whole-cell increase in synaptic strength observed after learning the task is not the result of homeostatic synaptic scaling [42, 43]; Learning-induced increase in synaptic strength occurs when the spike firing rate is enhanced [44–46] rather than decreased [42,43]. Thus, while homeostatic synaptic scaling is a negative feedback control mechanism, learning-induced whole-cell synaptic strengthening appears to be a positive feedback mechanism. Moreover, while the memory amplification mechanism appears to induce a uniform scaling of all synapses, homeostatic synaptic scaling appears to cause a general increase in synaptic strength which does not appear to be uniform. Indeed, increase in synaptic strength that is supported by a pseudo binary increase in single channel conductance as we showed for the memory amplification mechanism allows such a uniform increase, while increase in synaptic strength which is supported by addition of synaptic receptors appears to be more general and less uniform [43].

An across-the-board increase in the strength of all excitatory synapses in a sub-group of cells cannot be explained by the classical learning theories that underlie memory formation in which only synapses relevant to the storage of a memory are strengthened. Moreover, while we show that doubling the channel conductance underlies the increase in synaptic strength; activity-dependent synaptic enhancement is thought to be mediated by an increase in the number of AMPA channel receptors [47, 48]. Indeed, memory amplification should not modify the composition of the subset of cells that compose the memory and thus should be uniformly applied on all synapses in this subset. Moreover, since memory formation is tuned by the correlation between presynaptic and postsynaptic activity in each synapse, it should support a continuous increase in synaptic strength and thus can be implemented by addition of AMPA channels. In contrast, a linear amplification of a memory can be induced by multiplying its firing response by a single factor and thus can be implemented by a uniform two-fold increase of the synaptic strength.

We presented a cell-specific amplification mechanism that can induce a memory selective amplification process when induced on a subset of cells that comprise a memory-pattern. This memory enhancement is independent of memory formation. A memory of vital importance needs to be enhanced to dominate subsequent behavior [49, 50]. When the memory becomes less crucial it should be de-enhanced to balance its weight with the weights of other memories. The induction of whole cell multiplication via a whole cell transduction mechanism and its action through channel conductance enables it to be readily toggled-on when needed and off when no further needed. Such properties may underlie the extinction and relapse seen in our behavioral settings. Thus whole-cell CaMKII mediated phosphorylation can function as a “software switch” unlike a hardware switch that requires morphological modulation as was suggested in processes that accompanies memory formation.

## Materials and Methods

### Experimental procedures

In this study we have used data from previously published experiments in which training- induced modification of inhibitory [8] and excitatory [7] synaptic transmission was studied. For sake of clarity we present here a brief description of the animal training and the experimental recording setup. More details can be found in previously published works (behavioral settings: [1, 2,51]; Slice preparation and recordings: [7,8])

Animal training: Rats designated to the trained group were rewarded with water when they chose the correct positive cue. Rats designated to the pseudo-trained group were rewarded with water when choosing any odor in a random manner, and rats in the naive group were left in their home cages and were water deprived only. Training consisted of 20 trials per day for each rat [2]. Two learning phases can be seen during training [1,6]: in the initial phase of "rule learning", which requires 7–8 consecutive training days, the rat forms a strategy for executing the olfactory discrimination task, and in the second phase the rat exhibits an enhanced learning ability, and can learn novel odors within 1–2 training days. Once the rats have reached the criterion for olfactory discrimination, they were allowed to rest for 4–5 days and then brain slices were prepared.

Recordings [7, ‎8]: Whole cell recordings of spontaneous events where done in the presence of 1 μM tetrodotoxin and were performed 10–15 min after membrane rupture and lasted for up to 45–50 min. Miniature excitatory post-synaptic currents (mEPSCs) were recorded at holding potential of −80 mV causing miniature inhibitory synaptic events to be exceedingly small due to the small driving force and leaving most voltage-dependent channels closed and NMDA receptors seldom activated. To record GABA_A_-mediated miniature IPSCs (mIPSCs), the recording electrode contained 140 mM cesium chloride solution in order to reach a reversal potential of 0 mV, yielding a strong GABA_A_-mediated currents at holding potential of −60 mV. The perfusion solution also contained DNQX (20 μM) and APV (50 μM), to block glutamatergic synaptic transmission via AMPA receptors, thus allowing recording of pure IPSCs.

CaMKII blockers: the CaMKII inhibitor KN93 (10 μM) and the CaMKII cell-penetrating peptide inhibitor, tatCN21 (5 μM; GL Biochem, Shanghai Ltd, China) were used in order to inhibit the active state of CaMKII.

### Analyzing single channel current

Estimate of the average single channel current and the average number of active channels were obtained using a peak-scaled non-stationary fluctuation analysis (NSFA) of synaptic events [13]. The NSFA was applied on the events that were electrotonically nearby (10–90% rise-times < 1.5 ms). Using Mini analysis software (Synaptosoft Inc.), events (80–300 per each cell) were scaled and aligned by their peak, and their decay phase was divided to 30 bins. The single channel current (i) and the number of channels (N) were calculated (using Mini Analysis Software) by fitting the theoretical relationship for the peak scaled variance (*σ*^2^) after subtraction of the background variance $\sigma_{b}^{2}$:

$$\sigma^{2}\;(t)=i\cdot I(t)–I{\left(t\right)}^{2}/N+\sigma_{b}^{2}$$

Only cells in which the fitting of the equation yielded an R>0.85 were incorporated in the analysis.

### Analyzing the fraction of events affected by the blocker

Under the assumption of a model in which the blocker effect (averaged events amplitude before the blocker divided by the averaged events amplitude after the blocker) on the event amplitude is a division by a factor of ***a***, given that a fraction ***x*** of the events was affected by the blocker then the blocker effect ***y*** should be calculated as follows:

y=(1−x)+x*a

Some algebra enables as to calculate the fraction of the events that was modified by the blocker (**x)** as

$$x=\frac{y-1}{a-1}$$

### Modeling

This study examined the effect of whole-cell balanced multiplication on the spiking activity of neurons, using computer simulations. We used a conductance based cell with simplified morphology and a realistic generation and adaptation of action potentials as was observed in recordings from pyramidal cells in the piriform cortex [2].

### Morphology and passive properties

The uniform whole-cell property where the strength of all synapses is doubled makes whole-cell balanced amplification relatively insensitive to the locality of the synaptic responses and thus relatively independent of the precise morphology of the cells. This enabled us to implement the modeled cell morphology as a simplification of a pyramidal cell where the apical and basal dendritic trees were reduced to long cylinders connected to the soma [52]. Both the morphology and the passive properties were constructed such that the membrane decay time constant (12 ms) and the size of a single excitatory synaptic current (1 mV) would approximate previously measured parameters in-vitro [1, 53]. In our model cell, the input resistance (114 MΏ) was in the high range of previously reported values [1], in order to compensate for the leak introduced by the sharp electrode used in these experiments.

### Synaptic input

The currents generated by the excitatory and inhibitory synapses were modeled by an alpha function with realistic rise times and decay times. The excitatory and inhibitory synapses had characteristics of decay and rise times as measured for AMPA and GABA_A_ receptor mediated currents [6]. 350 Excitatory and 350 inhibitory synapses were uniformly distributed along the apical and basal dendrites. The reversal potential of the net synaptic current resulted to a value of around -37mV, which similar to the value measured experimentally during UP-states [20], and is theoretically expected at balanced state. In our simulations, we measured the reversal potential of the net synaptic current by measuring the amplitudes of the averaged net synaptic current at various holding potentials.

The amplitudes of the excitatory synapses were chosen based on the excitatory miniature event amplitude distribution (Fig 5B, naive) and scaled to result in average amplitude of 70pA. The scaling factor reflects the ratio of the average miniature excitatory synaptic event (mepsc) amplitude and the average synaptic response to one presynaptic action potential in piriform neurons [54]. Since there is no definitive evidence that in general Hebbian learning also modifies the inhibitory synapses, for simplicity we modeled the inhibitory synapses as a uniform distribution where the average response yielded a synaptic current with amplitude of 100 pA. The activation frequencies of inhibitory and excitatory synapses differed across simulations and ranged from 2 to 5 Hz. These values resulted in a voltage standard deviation of 3.9±0.23 mV.

The reversal potential of the net synaptic current was determined as the holding potential at which the net synaptic current averaged over 200 ms was amounted to zero.

### Varying proportions of strong excitatory synapses

Although the tail of this distribution cannot be fully accounted by learning, a simple way to divide the population of synapses to strong and weak synapses is through a division of the miniature amplitude distribution curve. The synapses were divided into strong and weak based on the miniature amplitude distribution curve (Fig 5A, weak synapses < 13 pA the Gaussian like section of the distribution; strong synapses > = 13 pA the tail of the distribution). When scaling values between zero and one, strong synapses had a relative conductance of 0.25–1 and weak synapses had a relative conductance of 0.04–0.25. The rationale behind increasing the proportion of strong synapses is that in Hebbian learning synapses that connect co-active cells become stronger. Therefore when a cell participates in a learned Hebbian memory the proportion of strong synapses will increase.

We divided the increase into four levels where the proportion of active strong excitatory synapses was 30%, 45% and, 60%. At rest, strong and weak synapses had the same activation probability. The increase in the proportion of strong synapses was generated by increasing the frequency of the strong synapses and decreasing the frequency of weak synapses. The decrease in the frequency of weak synapses and the increase in the frequency of strong synapses were done such that the total number of excitatory synaptic events was maintained constant.

### Active conductance

The whole-cell balanced amplification mechanism acts directly through modulation of the synaptic strength and is only indirectly dependent on intrinsic excitability. In this paper we aimed to understand the functional outcome of this mechanism and not address its quantitative effect on spike frequency. Therefore we did not do extensive modeling of the cell’s intrinsic properties. We modeled a regular spiking pyramidal cell that exhibits moderate adaptation [2]. We included voltage dependent currents that are known to participate in the generation of the action potential and the refractory period: transient (inactivating) sodium current g_Na_; delayed rectifier potassium current g_K_(DR); transient inactivating g_K_(A); In addition we included currents that participate in spike adaptation: muscarinic receptor suppressed potassium conductance g_K_ (M); slow calcium dependent potassium conductance g_K_ (AHP). For the activation of the g_K_ (AHP) we implemented a high threshold non- inactivating calcium conductance g_Ca_(H) and calcium homeostasis mechanism. [Ca]_i_ was elevated by the calcium current and decayed due to calcium homeostasis mechanisms according to the following equation:

$${\left[Ca\right]}_{i}^{\prime }=\frac{-I_{ca}}{depth∙F∙4000}∙10^{7}-\frac{{\left[Ca\right]}_{i}-{\left[Ca\right]}_{o}}{\tau },$$

where depth is the cell diameter, and τ is the time constant of [Ca]_i_ removal.

The equation describing muscarinic conductance was taken from [55]; the equations describing the rest of the active conductance were taken from [56] in a work that modeled a cortical pyramidal cell. The rate parameters were kept at the same values and conductance densities were varied to result in spiking activity that resembled a regular spiking pyramidal cell. The conductances that generate the action potential (g_Na_; g_K(DR_; g_K(A)_) were located in the soma whereas the currents that underlie the after- hyperpolarization and the calcium current (g_k(M)_; g_ca(H)_; g_k(AHP_)) were located in the proximal apical dendrite.

The response of the cell to a step current was similar to the responses of cells as recorded in the piriform cortex, both in the characterization of action potential and their firing frequency adaptation (Fig 2A; [1, 2]).

### Whole-cell balanced multiplication

The mechanism of whole-cell balanced multiplication was implemented by multiplying the strength of all excitatory and inhibitory synapses. As was observed previously (see Results), the increased strength was mediated by multiplying the conductance of both the excitatory and the inhibitory synapses by a factor of 2.

### Simulation parameter space

The default values for the simulation parameters are noted at Table 1. The parameter space for Fig 7 was created by varying the amplitudes of the excitatory and inhibitory currents and their activation frequency. The average conductance of the AMPA receptor ranged from 0.6 and 10 nS and the average conductance of the GABA_A_ ranged from 0.9–2.7 nS. For each value of the excitatory and inhibitory conductance the activation frequency of both synapses ranged from 2-5Hz. Only simulations that yielded an average membrane voltage of less than -55 mV were included.

**Table 1:** Values for simulation parameters.

Morphology and passive properties	
Length/diameter of apical dendrite	200/2.5 μm
Length/diameter of basal dendrite	80/3 μm
Length/diameter of soma	12/20 μm
Axial resistivity (Ra)	50 Ώ cm
Membrane capacitance	2 μF/cm^2^
Leak conductance	0.0002 S/cm^2^
Leak reversal potential	-80 mV
**Active conductance**	
g_na_	0.2 S/cm^2^
g_k(DR)_	0.14 S/cm^2^
g_k(A)_	0.1 S/cm^2^
g_k(M)_	0.01 S/cm^2^
g_ca(H)_	0.003 S/cm^2^
g_k(AHP)_	0.002 S/cm^2^
Sodium reversal potential	55 mV
Potassium reversal potential	-90 mV
Calcium reversal potential	90 mV
τ of [Ca]_i_ homeostasis	10 ms

For each point in this parameter space, simulations were performed for the 4 activation levels of the strong synapses; for each level the average over ten trials was calculated and each trial was calculated over a period of 500ms. In this parameter space where both the average amplitude and activation frequency of the synapses varied, the net synaptic current before whole-cell balanced amplification was used as a uniform indicator of the synaptic strength.

For Figs 9 and 10 the parameter space was created by varying the reversal potential of the leak current from -100 to -70. This manipulation modified the averaged membrane potential during baseline activity from -62 mV to -75 mV, which effectively shifted the distribution of the baseline membrane potential relative to threshold and is equivalent to modification of the term $\frac{\theta }{\sigma }$ in Eq 2. In addition, the strength of the excitatory and inhibitory synapses was varied by independent multiplication by factors that ranged from 0.6 to 2, and the activation frequency of the inhibitory and excitatory synapses was co-modulated up to a factor of 3. These manipulations are equivalent to modulating ***σ*** and $\bar{I}$.

As the aim of these simulations was to yield instantaneous and not the average effect of whole cell multiplication, the results presented in this figure are from single traces.

Simulations were conducted using the Neuron simulation program [57].

## Supporting Information

S1 Text (DOCX)

S2 Text (DOCX)
