# Supplementary material for: Real Time Multiplicative Memory Amplification Mediated by Whole-Cell Scaling of Synaptic Response in Key Neurons
Source: PLoS Comput Biol. 2017 Jan 19;13(1):e1005306. doi: 10.1371/journal.pcbi.1005306 (PMC5245787; doi:10.1371/journal.pcbi.1005306)
Supplement: S1 Text — (DOCX) [file pcbi.1005306.s001.docx]

Appendix 1

We assumed that the synaptic current had an instantaneous rise time and an exponential decay. In addition we assumed that the synaptic events were independent; hence the total variance can be calculated by summation of the variance added by each synaptic event. The variance added by each synaptic event was calculated as follows:

$$\boldsymbol{\sigma}^{\mathbf{2}}=\int_{\mathbf{0}}^{\boldsymbol{T}} \frac{{(\boldsymbol{\omega}\cdot\boldsymbol{e}^{-\frac{\boldsymbol{t}}{\tau}}- \boldsymbol{\mu})}^{\mathbf{2}}}{\boldsymbol{T}}\boldsymbol{dt}$$

where τ is the decay time constant of the exponent, **T** is the time length in which the variance is calculated and is assumed to be >>τ, ω is the initial amplitude of the exponent and is assumed to be uniform over all epsc's and **μ** is the average synaptic current, which can be calculated as follows:

$\boldsymbol{\mu}=\boldsymbol{\omega}\cdot\frac{\int_{\mathbf{0}}^{\boldsymbol{T}} \boldsymbol{e}^{-\frac{\boldsymbol{t}}{\tau}}}{\boldsymbol{T}}$ $=\boldsymbol{\omega}\cdot\frac{\tau}{\boldsymbol{T}}$

With **N** synaptic events the total variance is:

$$\boldsymbol{\sigma}^{\mathbf{2}}=\boldsymbol{N}\cdot\int_{\mathbf{0}}^{\boldsymbol{T}} \frac{{(\boldsymbol{\omega}\cdot\boldsymbol{e}^{-\frac{\boldsymbol{t}}{\tau}}- \boldsymbol{\mu})}^{\mathbf{2}}}{\boldsymbol{T}}\boldsymbol{dt}$$

$$=\boldsymbol{N}\cdot\boldsymbol{\omega}^{\mathbf{2}}\cdot\left( \frac{\boldsymbol{\tau}}{\mathbf{2}\boldsymbol{T}}-\frac{\boldsymbol{\tau}^{\mathbf{2}}}{\boldsymbol{T}^{\mathbf{2}}} \right)$$

This equation can be expanded to calculate the variance when the amplitude of the epsc's is not uniform. The variance of events’ amplitude is calculated as the square of the coefficient of variance (CV) multiplied by the averaged amplitude ($\bar{\omega}$). The total variance is calculated as a summation of the variance created by the addition of each synaptic event and the variance of events’ amplitudes:

$\boldsymbol{\sigma}^{\mathbf{2}}=\boldsymbol{N}\cdot\left\{ \int_{\mathbf{0}}^{\boldsymbol{T}} \frac{{(\bar{\boldsymbol{\omega}}\cdot\boldsymbol{e}^{-\frac{\boldsymbol{t}}{\tau}} - \boldsymbol{\mu})}^{\mathbf{2}}}{\boldsymbol{T}}\boldsymbol{dt}+ \left( \boldsymbol{CV}\cdot\boldsymbol{\mu} \right)^{\mathbf{2}} \right\}$,

where $\bar{\omega}$ is the average epsc amplitude and CV is the coefficient of variation of the epsc amplitudes,

$$={\bar{\boldsymbol{\omega}}}^{\mathbf{2}}\cdot\boldsymbol{N}\cdot\frac{\boldsymbol{\tau}}{\boldsymbol{T}}\cdot\left( \frac{\mathbf{1}}{\mathbf{2}}+\frac{\tau}{\boldsymbol{T}}\cdot\left( \boldsymbol{CV}^{\mathbf{2}}-\mathbf{1} \right) \right)$$
