# Supplementary material for: Real Time Multiplicative Memory Amplification Mediated by Whole-Cell Scaling of Synaptic Response in Key Neurons
Source: PLoS Comput Biol. 2017 Jan 19;13(1):e1005306. doi: 10.1371/journal.pcbi.1005306 (PMC5245787; doi:10.1371/journal.pcbi.1005306)
Supplement: S2 Text — (DOCX) [file pcbi.1005306.s002.docx]

# Appendix 2

We assumed a neuron with a threshold linear transfer function $H\left( x \right)$ that obeys:

$$H(x)= \left\{ \begin{aligned} x\geq\theta\beta(x-\theta) \\ x<\theta0 \end{aligned} \right.$$

where x is the net synaptic current, $\theta$ is the synaptic current required to reach threshold.

When assuming that the fluctuations in the net synaptic current obeys Gaussian statistics the average firing rate as a function of the average net synaptic current can be evaluated using the following equation:

$$F(\overline{I}, \sigma)=\int_{-\infty}^{\infty} p\left( h \right)\cdot H\left( \overline{I}+\sigma h \right)dh$$

where $p(h)$ is the probability of having a fluctuation noise of size $h$ assuming Gaussian statistics with width 𝜎 in the current (${\frac{1}{\sqrt{2\pi}}e}^{-x^{2}/2}$).

$$F(\overline{I}, \sigma)=\int_{\frac{\theta-\overline{I}}{\sigma}}^{\infty} \frac{1}{\sqrt{2\pi}}e^{-h^{2}/2}\cdot\left( \overline{I}+\sigma h-\theta\right)\cdot\beta dh$$

Splitting this expression to a summation of two integrals leads to:

$$= \beta\cdot\left( \frac{1}{\sqrt{2\pi}}\int_{\frac{\theta-\overline{I}}{\sigma}}^{\infty} e^{{-h}^{2}/2}\cdot\sigma h dh+\left( \bar{I}-\theta\right)\cdot\frac{1}{\sqrt{2\pi}}\int_{\frac{\theta-\overline{I}}{\sigma}}^{\infty} e^{-h^{2}/2}dh \right)$$

Solving the right hand integral and changing the order of the differential and the integral operator in the left hand expression yields to:

$$= \beta\cdot\left( -\frac{\sigma}{\sqrt{2\pi}}\frac{\partial\int_{\frac{\theta-\overline{I}}{\sigma}}^{\infty} e^{-\frac{h^{2}}{2}}dh}{\partial h} +\left( \bar{I}-\theta\right)\cdot\frac{1}{2}\cdot\left( 1-erf\left( \frac{\theta-\overline{I}}{\sigma\sqrt{2}} \right) \right) \right)$$

Solving the left hand expression leads to:

$$= \beta\cdot\sigma\left( \frac{1}{\sqrt{\pi}}\cdot e^{{-\left( \frac{\overline{I}-\theta}{\sigma\sqrt{2}} \right)}^{2}} + \frac{\left( \bar{I}-\theta\right)}{2\sigma}\cdot\left( 1+erf\left( \frac{\overline{I}-\theta}{\sigma\sqrt{2}} \right) \right) \right)$$
